# Roles of gut microbiota in androgenetic alopecia: insights from Mendelian randomization analysis

**DOI:** 10.3389/fmicb.2024.1360445

**Published:** 2024-04-02

**Authors:** Haijing Fu, Tianyi Xu, Wumei Zhao, Leiwei Jiang, Shijun Shan

**Affiliations:** ^1^Department of Dermatology, Xiang’an Hospital of Xiamen University, School of Medicine, Xiamen University, Xiamen, China; ^2^Department of Dermatology, Guizhou Provincial People’s Hospital, Guiyang, China; ^3^Hangzhou Third People’s Hospital, Affiliated Hangzhou Dermatology Hospital, Zhejiang University School of Medicine, Hangzhou, China

**Keywords:** androgenetic alopecia, causal relationship, genetic, gut microbiota, Mendelian randomization

## Abstract

**Background:**

Androgenetic alopecia (AGA) is the most common type of androgen-associated hair loss. Previous studies have indicated an association between the gut microbiota and AGA. To delve deeper, we executed a two-sample Mendelian randomization (MR) analysis to investigate the potential causal relationship between the gut microbiota and AGA.

**Methods:**

A two-sample MR investigation was utilized to delve into the intricate interplay between gut microbiota and AGA. Information regarding 211 gut microbial taxa was sourced from the MiBioGen consortium. The summary statistics of the genome-wide association studies (GWAS) for AGA were obtained from the FinnGen biobank, which included 195 cases and 201,019 controls. Various analytical approaches, including Inverse Variance Weighting (IVW), Weighted Median, MR-Egger, Weighted Mode, and Simple Mode were employed to evaluate the causal impact of gut microbiota on AGA. Sensitivity analyses were subsequently conducted to affirm the robustness of the findings.

**Results:**

A two-sample MR investigation unveiled the genus *Olsenella*, genus *Ruminococcaceae UCG-004*, and genus *Ruminococcaceae UCG-010* were identified as risk factors associated with AGA. In contrast, the family *Acidaminococcaceae* and genus *Anaerofilum*, along with the genus *Ruminiclostridium* 9, demonstrated a protective effect. The sensitivity analyses provided additional assurance that the findings of the current study were less susceptible to the influence of confounding variables and biases.

**Conclusion:**

The MR study has established a link between specific gut microbiota and AGA, offering evidence for the identification of more precisely targeted probiotics. This discovery has the potential to aid in the prevention, control, and reversal of AGA progression.

## Introduction

Male Pattern Hair Loss (MPHL) and Female Pattern Hair Loss (FPHL) also referred to as Androgenetic alopecia (AGA) stand out as the most prevalent form of hair loss, impacting a substantial portion of the population. It is estimated that by the age of 70, at least 80% of men and 50% of women experience AGA (Devjani et al., 2023). AGA is marked by the gradual miniaturization of hair follicles, resulting in hair loss (Trüeb, 2002). Dealing with alopecia is a challenging and time-consuming process. Individuals experiencing hair loss often suffer from a diminished quality of life, including reduced self-confidence and heightened feelings of depression (Lee et al., 2002; Yeo et al., 2014; Marks et al., 2019). Consequently, the effective management of hair loss plays a crucial role in improving people’s overall wellbeing. To enhance our ability to prevent and treat AGA effectively, it is essential to acquire a more comprehensive understanding of the underlying mechanisms that contribute to its development. Nevertheless, the precise cause behind the escalating incidence rate of AGA has yet to be fully elucidated.

Numerous factors influence the initiation and progression of AGA, the interaction of endocrine factors and genetic predisposition is one of the primary factors (Lolli et al., 2017). Studies have confirmed that a range of external factors, such as metabolism, psychological changes, environmental exposure, dietary intake, and microorganisms, can have adverse effects on the lifespan of hair (Lai et al., 2013; Phillips et al., 2017; Ho et al., 2019; Suzuki et al., 2021). Recently, there is a study has shown that gut microbiota also is an essential factor in the development of AGA (Jung et al., 2022). However, in research related to AGA, there is a relatively limited study on the specific role of the gut microbiome in AGA. The conventional observational study is susceptible to the impact of numerous potential factors, including lifestyle and socioeconomic status, during the implementation process, rendering it prone to biases. Hence, we examined existing summary data from the results of genome-wide association studies (GWAS) to investigate the influence of gut microbiota on AGA.

Genome-wide association studies with large sample sizes has revealed some single nucleotide polymorphisms (SNPs) correlated with both AGA and gut microbiota (Wang et al., 2019). Mendelian randomization (MR) is a method that employs genetic variants linked to a hypothesized risk factor as proxies to ascertain the causal impact of that exposure on a specific outcome (Birney, 2022). In this study, we evaluated the causal effects of gut microbiota and AGA using a two-sample MR study design. Our results demonstrated a potential causal association between specific gut microbiota and AGA.

## Materials and methods

### Study design

**FIGURE 1:**
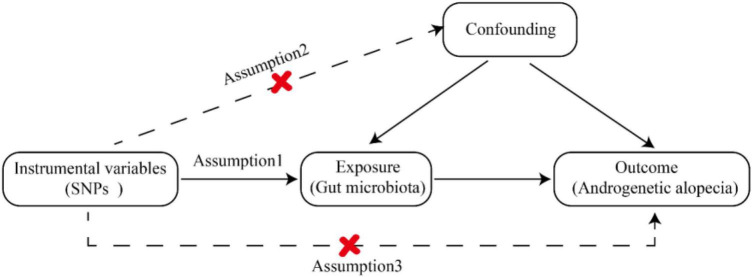
Comprehensive flowchart of study.

To explore the interaction between gut microbiota and AGA, we designated gut microbiota as the exposure variable, with AGA considered as outcomes. The MR study (Evans and Davey Smith, 2015) adhered to three crucial assumptions: (1) Instrumental variables (IVs) chosen from datasets were connected to the exposure variable; (2) they were independent of any unknown confounders related to the exposure; and (3) they exclusively influenced outcomes through exposure pathways (Davey Smith and Hemani, 2014). SNPs were employed as valid IVs in the MR study to assess the bidirectional causal relationship between the exposure and the outcome. The comprehensive flowchart for this MR study is illustrated in Figure 1.

### Data sources

Instrumental variables (IVs) for investigating the correlation between human genetic variants and the composition of the gut microbiome were identified from a GWAS dataset within the global collaborative project MiBioGen (Kurilshikov et al., 2021). This extensive, multi-ethnic GWAS involved the coordination of 16S ribosomal RNA gene sequencing profiles and genotyping data from 18,340 participants. A total of 211 taxa, comprising 131 genera, 35 families, 20 orders, 16 classes, and 9 phyla, were encompassed in the analysis (Kurilshikov et al., 2021; Cui et al., 2023). The dataset for AGA included 201,214 European participants sourced from the freely accessible website FinnGen biobank.^1^ The specific has been placed in the Supplementary Table 4.

### Instrument variable selection

We implemented a set of criteria to carefully select eligible genetic IVs:(1) Significance Threshold: Due to the limited number of IVs meeting the genome-wide significance threshold (*p* < 5 × 10^–8^) (Kurilshikov et al., 2021; Herbert et al., 2022), we opted for a relatively less stringent threshold (*p* < 1 × 10^–5^) based on previous research (Cui et al., 2023; Dai et al., 2023; Liu et al., 2023; Xiao et al., 2023). This less stringent threshold was selected to identify potential sets of variants that are likely to be enriched for association, allowing for a more comprehensive assessment and exploration of results. (2) Clumping Procedure: A clumping procedure was executed (*R*^2^ < 0.001, clumping distance = 10,000 kb) to eliminate variants in strong linkage disequilibrium (LD). This step ensured the independence of each SNP in our selection. (3) To mitigate bias stemming from weak IVs, it was deemed necessary to ensure that the F-statistic surpassed a threshold of 10. This threshold helps assess the strength of the instruments and provides confidence in the instrumental variable analysis results (Ardissino et al., 2024).

### Assessment and diagnostic criteria for AGA

Clinical manifestations vary between genders. Males experience frontotemporal recession and vertex loss, while females retain the frontal hairline, with hair loss more uniformly across the frontal region post-hairline. Gender-atypical patterns can occur. Assessment of AGA commonly employs the Hamilton-Norwood scale (12 degrees), and the Ludwig scale (3 stages). Diagnostic criteria include miniaturized follicles (hair diameter <0.03 mm), decreased terminal and anagen hairs, increased vellus and telogen hairs, and perifollicular lymphohistiocytic inflammatory infiltrate around the infundibulum (Alessandrini et al., 2021).

### MR analysis

We utilized five extensively employed Mendelian Randomization (MR) techniques to identify bidirectional causal connections between exposure and outcome. These methods include inverse variance weighting (IVW), weighted median, MR-Egger, weighted mode, and simple mode (Bowden et al., 2016; Hartwig et al., 2017; Liu et al., 2023). The IVW method calculates the causal impact of the exposure on the outcome by aggregating ratio estimates for each SNP. It was selected as the primary method due to its capacity to offer a robust and unbiased causal effect, especially in scenarios where no polymorphism or heterogeneity is identified (Mounier and Kutalik, 2023). A positive causal effect was confirmed if the IVW results were significant (*p* < 0.05), and the beta values from other methods were consistent in direction. Subsequently, we proceeded to visually represent the outcomes derived from the five Mendelian Randomization (MR) methods. The bidirectional causal effect was quantified as an odds ratio (OR) computed through MR analysis.

To enhance the robustness of our analysis regarding causality, we employed the Bonferroni’s method to establish distinct significance thresholds for multiple testing across various taxonomic levels. These thresholds were determined based on the number of bacteria within each taxonomic level. Specifically, we set the thresholds at 1.6 × 10^–3^ (0.05 divided by 32) for the family level and 4.2 × 10^–4^ (0.05 divided by 119) for the genus level. *P*-values falling below these thresholds were considered to indicate nominal significance (*p* < 0.05), suggesting potential causal effects (Su et al., 2023). This study is reported following the Strengthening the Reporting of Observational Studies in Epidemiology Using Mendelian Randomization guidelines (STROBE-MR) (Skrivankova et al., 2021).

### Sensitivity analyses

Use some basic sensitivity analyses to validate the results. Cochran’s Q statistic is employed to assess heterogeneity among IVs (Bowden et al., 2018). A significance level of *p* < 0.05 was deemed indicative of the presence of heterogeneity. MR pleiotropy residuals and outlier analysis (MR-PRESSO) are also used to validate the potential pleiotropy of the selected IVs and the direct effects on outcomes. Subsequently, we apply the leave-one-out method to exclude each SNP from the IVs and use the IVW method to assess whether individual SNPs significantly affect the causal effect. All the aforementioned analyses are conducted using the MR and MR-PRESSO R packages with two-sample MR (Hemani et al., 2018; Verbanck et al., 2018).

We utilized GWAS Catalog^2^ to investigate whether SNPs were linked to potential risk factors. The factors encompass body mass index, obesity, alcohol consumption and neuropsychiatric diseases (Severi et al., 2003). We excluded SNPs associated with any of these potential confounders at genome-wide significance.

### Power calculation

The power calculation for the IVW estimates was conducted using a web tool available at https://shiny.cnsgenomics.com/mRnd/. A further explanation of power calculation can be seen in “binary outcome derivations” in the web tool and the type I error rate (alpha) is set at 0.05 (Brion et al., 2013). Specifically, it was recommended to achieve a statistical power of over 80% for the analysis (Zhong et al., 2023).

## Results

### MR analysis of gut microbiota on AGA

**FIGURE 2:**
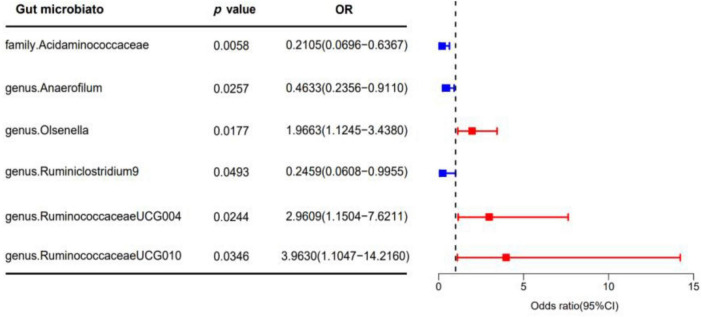
The forest plot illustrates the causal effect of gut microbiota on AGA using the IVW method in MR. The error bars in the plot represent the 95% confidence interval of the odds ratio. In the plot, the blue dots represent outcomes related to AGA, while the red dots represent outcomes associated with AGA positivity. OR, odds ratio; MR, Mendelian randomization; IVW, inverse variance weighted.

**FIGURE 3:**
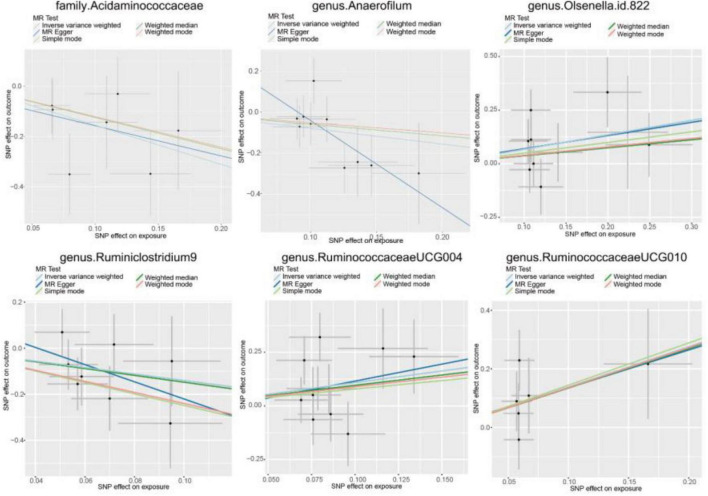
Scatter plots for causal effect of gut microbiota on AGA.

In our analysis, 62 SNPs were used as IVs for AGA. The comprehensive characteristics of these IVs pertaining to microbial taxa were summarized in Supplementary Table 1. Notably, all SNPs included in our analysis exhibited F-statistics greater than 19 indicating the robustness of these instruments. Upon reviewing the GWAS Catalog, we observed that only one SNP exhibits pleiotropic effects, primarily linked to smoking. Previous research indicates no significant association between AGA and smoking (Kavadya and Mysore, 2022). Therefore, we exclude the confounded without removing the SNP. MR analysis was conducted to evaluate the potential causal relationship between specific categories of gut microbiota and the occurrence of AGA. There are 6 bacterial taxa had a correlated with AGA. According to the IVW estimate, the genus *Olsenella* (OR = 1.9663, 95% CI: 1.1245–3.4380, *p* = 0.0177), genus *Ruminococcaceae UCG-004* (OR = 2.9609, 95% CI: 1.1504–7.6217, *p* = 0.0244), and genus *Ruminococcaceae UCG-010* (OR = 3.9630, 95% CI: 1.1047–14.2160, *p* = 0.0346) were identified as risk factors, (Figure 2) suggesting a potential association with decreased hair growth function. On the other hand, the family *Acidaminococcaceae* (OR = 0.2105, 95% CI: 0.0696–0.6367, *p* = 0.0058) and genus *Anaerofilum* (OR = 0.4633, 95% CI: 0.2356–0.9110, *p* = 0.0257), along with the genus *Ruminiclostridium 9* (OR = 0.2459, 95% CI: 0.0608–0.9955, *p* = 0.0493), demonstrated a protective effect, (Figure 2) implying a potential association with a reduced risk of AGA. Additionally, the estimates of causal effects obtained from the weighted median, MR-Egger, weighted mode, and simple mode methods demonstrated magnitudes and directions that were comparable to those derived from the previously mentioned IVW method, as detailed in Supplementary Table 2 and Figure 3. These findings provide insights into the specific gut microbiota components that may play a role in the development of AGA and its potential link to AGA.

### Sensitivity analysis

**FIGURE 4:**
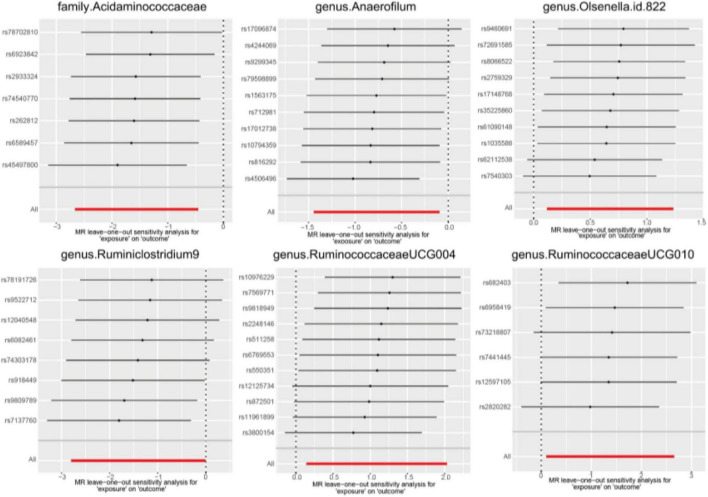
Leave-one-out diagrams for causal effects of gut microbiota on AGA.

In the sensitivity analysis, we employed Cochran’s Q statistics with both IVW and MR-Egger methodologies to evaluate heterogeneity. The results indicated no significant heterogeneity among the IVs, as evidenced by all *p-*values > 0.05 (Supplementary Table 3). Furthermore, both the MR-Egger intercept and the MR-PRESSO global test provided supporting evidence for the absence of statistically significant directional horizontal pleiotropy (all *p-*values > 0.05, Supplementary Table 3). Additionally, the leave-one-out analysis demonstrated the lack of influential IVs as illustrated in Figure 4.

These sensitivity analyses, encompassing Cochran’s Q statistics, MR-Egger intercept, MR-PRESSO global test, and leave-one-out analysis, collectively underscored the robustness of the Mendelian Randomization findings in the two-sample analysis. Furthermore, the presentation of the funnel plot and forest plots indicating the reliability of the results (see Supplementary Figures 1, 2).

## Discussion

Androgenetic alopecia is characterized by a progressive thinning of hair in specific areas, influenced by genetic and hormonal factors (Kwack et al., 2023). Prior research has hinted at a connection between AGA and the microbiome (Jung et al., 2022), but much remains unknown about the genetic factors driving this relationship. We employed a bidirectional two-sample MR analytical method, drawing data from GWAS databases encompassing both gut microbiota and AGA. Our inquiry delved into the intricate action of gut microbiota on AGA, unearthing significant insights into how gut microbiota influences AGA. Specifically, our findings spotlight the protective role of certain microbial—such as family *Acidaminococcaceae*, genus *Anaerofilum*, and genus *Ruminiclostridium 9*—in AGA. Conversely, genera like genus *Olsenella*, genus *Ruminococcaceae UCG-004*, and genus *Ruminococcaceae UCG-010* were identified as risk factors associated with AGA.

The human microbiota has evolved in tandem with its host and serves as an essential component of the human body. Acquired at birth, the microbiota matures alongside the host and maintains significance throughout life, impacting various bodily functions from infancy to old age (Adak and Khan, 2019). Earlier investigations have indicated that in individuals with AGA, both the scalp and gut microbiomes exhibit greater complexity and density, as reflected by elevated values of network topological statistics such as degree centrality, vertices, and edges (Jung et al., 2022). Two patients with alopecia universalis experienced hair regrowth after undergoing fecal microbiota transplantation as a treatment for recurrent *Clostridioides difficile* infections (Rebello et al., 2017). Imbalanced gut microbiota, notably the proliferation of Lactobacillus murinus, followed by biotin deficiency, constitutes crucial factors contributing to the onset of alopecia (Hayashi et al., 2017). The transplantation of fecal microbiota or the use of a specific probiotic (*Bifidobacterium longum HK003*) obtained from the feces of healthy individuals could potentially stimulate hair regrowth (Lam et al., 2022). Concerning *Ruminiclostridium 9*, it has demonstrated its regulatory effects on lipid metabolism, inflammation reduction, enhancement of intestinal barrier function, weight gain reduction, and improved insulin sensitivity in mice, effectively countering obesity development (Wang et al., 2020). However, its specific role in AGA remains unstudied. Further research on the effect of the family *Acidaminococcaceae*, genus *Anaerofilum*, and genus *Ruminiclostridium 9* on AGA may guide targeted probiotic applications. Our findings suggest that modulating the gut microbiota through probiotics could offer new avenues for the prevention and treatment of AGA.

*Ruminococcaceae* is a family of bacteria that belongs to the *Bacillota* (formerly known as *Firmicutes*) which is one of the major bacterial phyla observed in the human microbiome. *Ruminococcaceae* is known to be a significant component of the gut microbiota and plays important roles in various physiological processes (Hou et al., 2022). *Ruminococcaceae* were good predictors of folliculitis decalvans (Moreno-Arrones et al., 2023). Patients undergoing finasteride treatment exhibited a reduction in *Ruminococcaceae* levels compared to a healthy control group (Diviccaro et al., 2019; Borgo et al., 2021). The You-gui pill could increasing *Ruminococcaceae UCG-007* and *Ruminococcaceae UCG-010* in the intestine, thereby aiding in the treatment of kidney-yang deficiency syndrome (Chen et al., 2019). In our research, we identified that genus *Ruminococcaceae UCG004* (with a statistical power of 98%) and genus *Ruminococcaceae UCG010* (with a statistical power of 100%) are implicated as risk factors in association with AGA. The power levels exceeding 80% for both underscore the reliability of the involvement of genus *Ruminococcaceae UCG004* and genus *Ruminococcaceae UCG010* in AGA. Additional investigation is required to delve into the role of *Ruminococcaceae* in AGA.

The gut microbiota comprises a unique blend of various organisms, including bacteria, viruses, archaea, protozoa, and fungi, which interact bidirectionally with the central nervous system, forming what is known as the microbiome-gut-brain axis (Mayer, 2011; Sharon et al., 2016). This axis encompasses immune, neural, endocrine, and metabolic pathways, with steroid hormones playing a significant role. Thus far, the steroidogenic capacity remains incompletely understood. However, microbial species such as *Clostridium scindens* have the potential to convert glucocorticoids into androgens (Ridlon et al., 2013; Ferreira-Halder et al., 2017), thereby fostering the development of AGA. Additionally, *Faecalibacterium* spp. is a notable producer of butyrate (Ferreira-Halder et al., 2017), a short-chain fatty acid that plays a crucial role in gut microbiota-brain communication (Dalile et al., 2019) and has recently been proposed to influence sleep modulation (Szentirmai et al., 2019). Consequently, *Faecalibacterium* spp. may be a potential target for therapy AGA. Interestingly, certain members of the human microbiota (e.g., *Bifidobacterium* spp. and *Lactobacillus* spp.) encode genes involved in GABA production, suggesting microbial involvement in the production of this neurotransmitter within the gut (Barrett et al., 2012).

The precise biological mechanisms by which microbiotas influence AGA development remain unclear. The microbiota plays a major role in the endocrine system by interacting with estrogen, androgens, insulin, and other hormones (Qi et al., 2021). The androgens are a critical factor that leads to the gradual conversion of terminal hairs into intermediate and vellus hairs, resulting in the gradual hair thinning and hair loss seen in patients with AGA (Lolli et al., 2017). Different types of gut microbiota can produce enzymes essential for androgen metabolism, facilitating the synthesis and alteration of androgens. The microbial-mediated degradation of testosterone has been witnessed across various environmental contexts. For instance, Actinobacteria and Proteobacteria possess the capability to break down androgens (Qi et al., 2021). Additionally, Clostridium scindens, harboring the 20α-hydroxysteroid dehydrogenase (HSDH) enzyme in its genome, is a human gut microbe with significant potential to convert glucocorticoids into androgens (Ridlon et al., 2013). Meanwhile, microbiota-host communication is primarily facilitated through secreted exosomes (Li Q. et al., 2019). Exosomes, extracellular vesicles involved in cell communication, homeostasis, differentiation, and organogenesis (Li et al., 2018, 2021; Lai et al., 2022), are pivotal in hair morphogenesis and regeneration, holding potential for alopecia treatment (Hu et al., 2020). Plasma exosomes, originating from host cells or gut microbiota, mediate local or remote mutual regulation (Li Y. et al., 2019; Li et al., 2020). Intriguingly, exosomes have been observed carrying Wnt proteins on their surface, inducing β-catenin activation—a crucial signaling pathway in hair morphogenesis and regeneration (Gross et al., 2012). Exosomes from adipose-derived stem cells positively impact hair regrowth by enhancing DPC proliferation via upregulating the Wnt/β-catenin, TNF-α signaling pathways, and vascular endothelial growth factor expression (Hu et al., 2020). Further investigation is required to explore the functions of the six microbiotas mentioned in our article on the composition and decomposition of androgens. Elucidating the potential biological mechanisms at microbiotas that might influence AGA development is crucial.

### Limitations

Following Bonferroni’s correction, no distinct causal relationship between gut flora, metabolites, and the risk factors associated with heart failure was identified. This implies that further investigations are essential to validate and confirm the potential relationships between these elements. The recommended threshold for statistical power is over 80%. Notably, the power values for genus *Olsenella* (93%), genus *Ruminococcaceae UCG004* (98%), and genus *Ruminococcaceae UCG010* (100%) all exceed 80%, thereby reinforcing the validity of our findings. However, it is noteworthy that the overall power calculated by our research institute is not entirely satisfactory. The power values for family *Acidaminococcaceae* (29%), genus *Anaerofilum* (41%), and genus *Ruminiclostridium 9* (31%) fall below the 80% threshold. This discrepancy might be attributed to the relatively small rate of cases/controls (195/201,019) in the GWAS data for AGA. To enhance the statistical power and obtain more accurate results in future studies, it is advisable to consider increasing the sample size of cases.

The systemic androgens and genetic factors are the primary causes of AGA, various external or exogenous factors also play a role in its development (Choi et al., 2017). Limited further studies due to inadequate summary data for various alopecia types, lack of stratification statistics for AGA degree, and absence of sex-based stratification data. Several limitations of our study exist. In conclusion, the intricate physiological mechanisms underlying the relationship between gut microbiota and AGA extend beyond the scope of our simplistic models. Subsequent research efforts should focus on identifying potential mechanisms to deepen our understanding of AGA for preventive measures.

## Conclusion

Our study pioneered a two-sample MR analysis using GWAS summary statistics to probe a potential causal connection between gut microbiota and AGA. This analytical approach not only holds promise for developing effective prevention and intervention strategies for AGA but also offers innovative insights into the underlying mechanisms of AGA through the lens of gut microbiota.

## Data availability statement

The original contributions presented in this study are included in the article/Supplementary material, further inquiries can be directed to the corresponding authors.

## Author contributions

HF: Conceptualization, Data curation, Investigation, Methodology, Project administration, Software, Validation, Visualization, Writing – original draft, Writing – review and editing. TX: Methodology, Writing – original draft. WZ: Methodology, Writing – original draft. LJ: Funding acquisition, Resources, Software, Writing – review and editing. SS: Funding acquisition, Resources, Supervision, Writing – review and editing.

## Footnotes

1
https://r9.finngen.fi/

2
https://www.ebi.ac.uk/gwas/
