# Supplementary material for: Roles of gut microbiota in androgenetic alopecia: insights from Mendelian randomization analysis
Source: Front Microbiol. 2024 Apr 2;15:1360445. doi: 10.3389/fmicb.2024.1360445 (PMC11018880; doi:10.3389/fmicb.2024.1360445)
Supplement: Supplementary file 2 [file Table_2.DOCX]

**Supplementary Material**


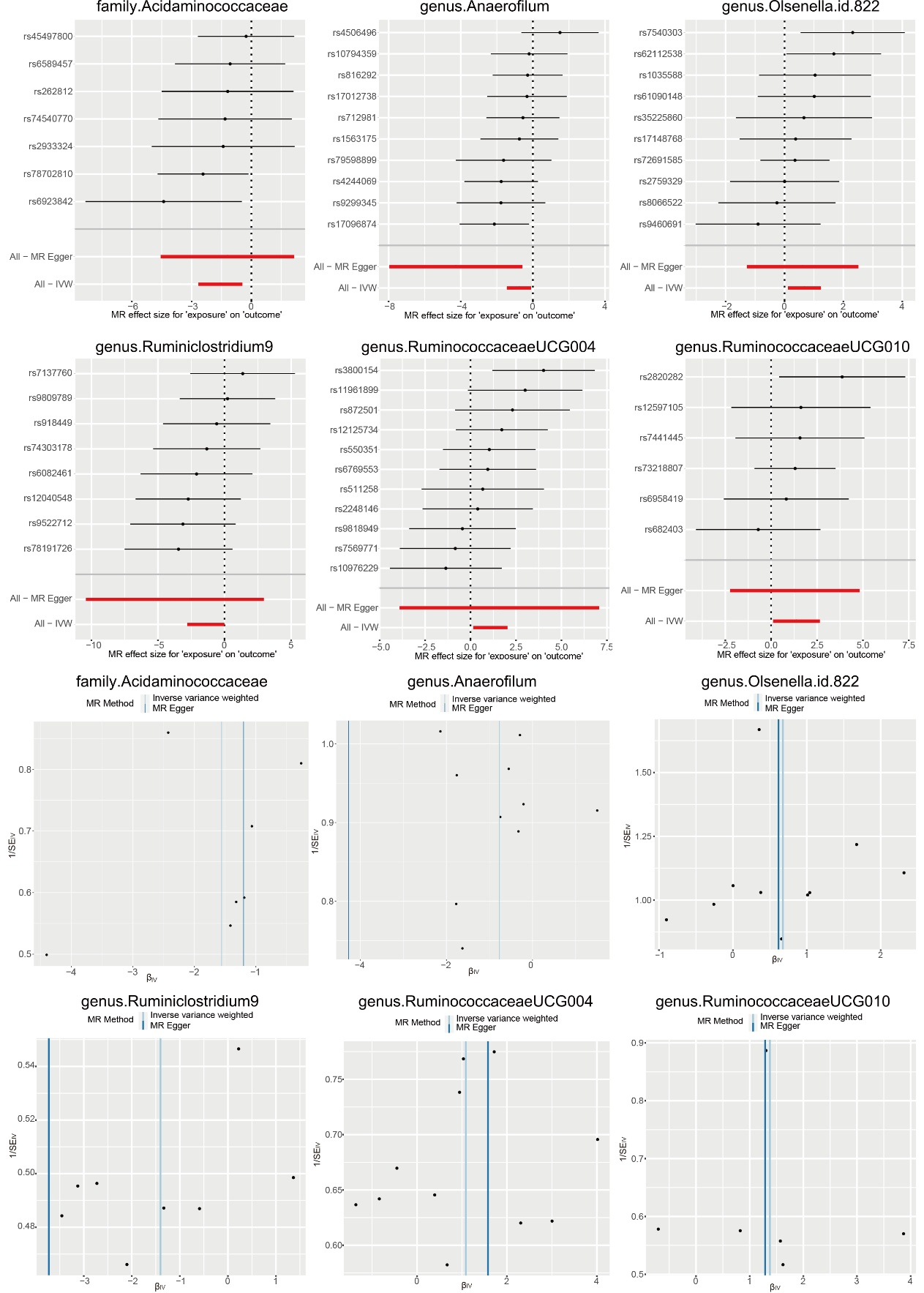


Figure S1: Forest plots for the effect of gut microbiota on AGA.


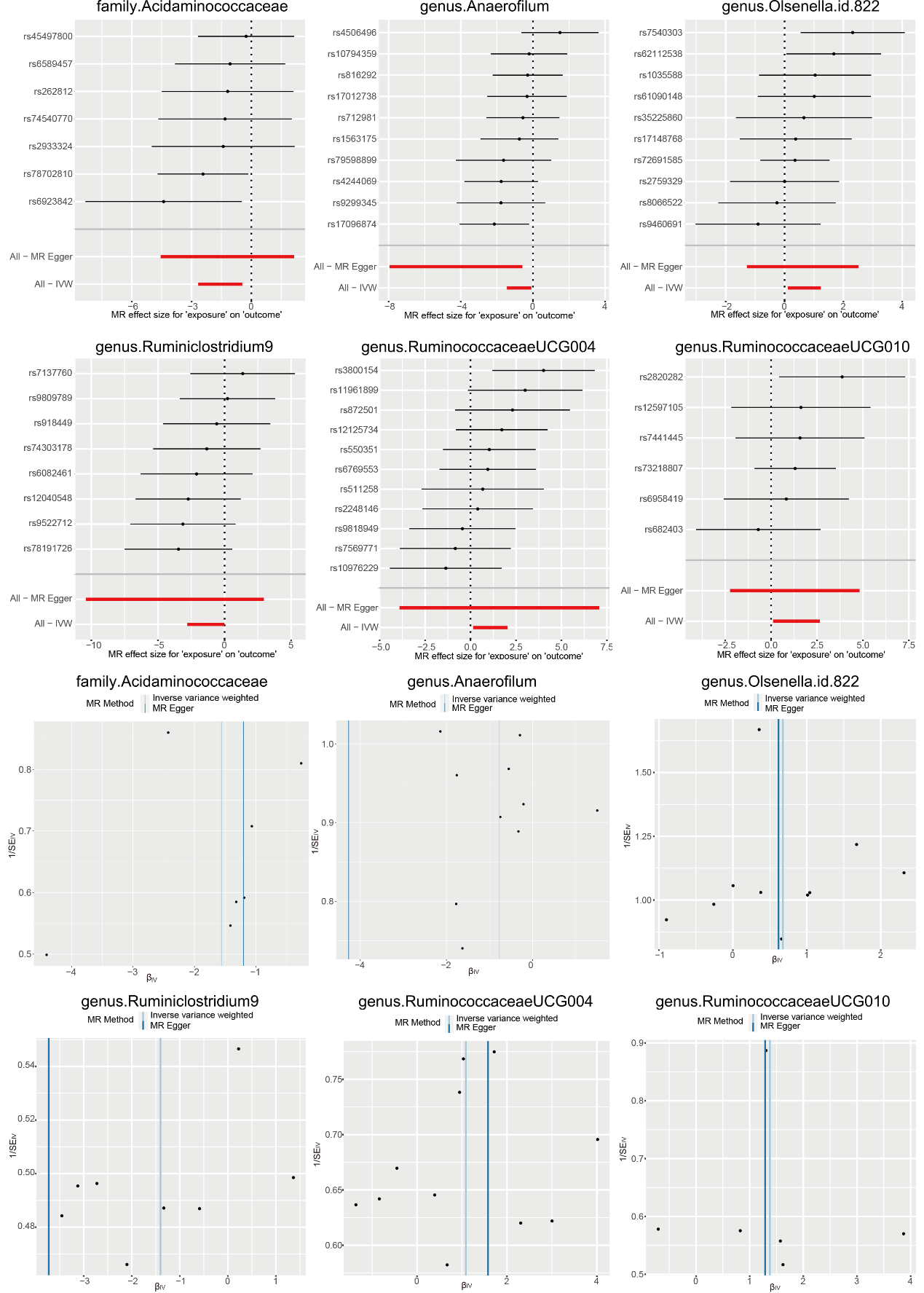


Figure S2: Funnel plot for the effect of gut microbiota on AGA.
